# The Evolutionary History of MAPL (Mitochondria-Associated Protein Ligase) and Other Eukaryotic BAM/GIDE Domain Proteins

**DOI:** 10.1371/journal.pone.0128795

**Published:** 2015-06-05

**Authors:** Jeremy G. Wideman, Blake P. Moore

**Affiliations:** 1 Department of Science, Augustana Faculty, University of Alberta, Camrose, Alberta, T4V 2R3, Canada; 2 Department of Cell Biology, Faculty of Medicine and Dentistry, University of Alberta, Edmonton, Alberta, T6G 2H7, Canada; Nazarbayev University, KAZAKHSTAN

## Abstract

MAPL (mitochondria-associated protein ligase, also called MULAN/GIDE/MUL1) is a multifunctional mitochondrial outer membrane protein found in human cells that contains a unique BAM (beside a membrane) domain and a C-terminal RING-finger domain. MAPL has been implicated in several processes that occur in animal cells such as NF-kB activation, innate immunity and antiviral signaling, suppression of PINK1/parkin defects, mitophagy in skeletal muscle, and caspase-dependent apoptosis. Previous studies demonstrated that the BAM domain is present in diverse organisms in which most of these processes do not occur, including plants, archaea, and bacteria. Thus the conserved function of MAPL and its BAM domain remains an open question. In order to gain insight into its conserved function, we investigated the evolutionary origins of MAPL by searching for homologues in predicted proteomes of diverse eukaryotes. We show that MAPL proteins with a conserved BAM-RING architecture are present in most animals, protists closely related to animals, a single species of fungus, and several multicellular plants and related green algae. Phylogenetic analysis demonstrated that eukaryotic MAPL proteins originate from a common ancestor and not from independent horizontal gene transfers from bacteria. We also determined that two independent duplications of MAPL occurred, one at the base of multicellular plants and another at the base of vertebrates. Although no other eukaryote genome examined contained a verifiable MAPL orthologue, BAM domain-containing proteins were identified in the protists *Bigelowiella natans* and *Ectocarpus siliculosis*. Phylogenetic analyses demonstrated that these proteins are more closely related to prokaryotic BAM proteins and therefore likely arose from independent horizontal gene transfers from bacteria. We conclude that MAPL proteins with BAM-RING architectures have been present in the holozoan and viridiplantae lineages since their very beginnings. Our work paves the way for future studies into MAPL function in alternative model organisms like *Capsaspora owczarzaki* and *Chlamydomonas reinhardtii* that will help to answer the question of MAPL’s ancestral function in ways that cannot be answered by studying animal cells alone.

## Introduction

Mitochondria are ubiquitous eukaryotic organelles derived from an ancient endosymbiotic α-proteobacterium. Tracing the evolutionary history of mitochondrial proteins is important in understanding how a free-living α-proteobacterium became an integrated eukaryotic organelle. Several mitochondrial proteins are closely related to α-proteobacterial proteins; however, it has been shown that many mitochondrial proteins are derived from bacteria not related to the endosymbiont [1,2]. The majority of the remaining mitochondrial proteins are eukaryote novelties that have no obvious homologues in bacteria [1,2]. To further complicate things, specific protein complexes are not either of endosymbiotic or eukaryotic origin, but instead, several systems like the mitochondrial import complexes and the complexes of the electron transport chain have mixed origins [2]. Some mitochondrial proteins have obscure evolutionary histories. The current study investigates the evolutionary origins of one such protein, the mitochondrial outer membrane protein MAPL (mitochondria-associated protein ligase, also called MULAN/GIDE/MUL1).

MAPL has been implicated in several processes specific to metazoan (multicellular animal) cells such as NF-kB activation, innate immunity and antiviral signaling, suppression of PINK1/parkin defects, mitophagy in skeletal muscle, and caspase-dependent apoptosis [3–6]. This, coupled with the fact that MAPL has homologues in α-proteobacterial, other bacteria, and archaea [7], calls into question the conserved ancestral function of MAPL. If MAPL is of endosymbiotic origin, then it likely has an ancient cellular function in addition to the aforementioned metazoan-specific functions. Indeed, other studies have uncovered MAPL’s role in potentially ancient processes like mitophagy, Akt regulation, regulation of mitochondrial dynamics, and sequestration as cargo in mitochondria-derived vesicles (MDVs) targeted to peroxisomes [6,8–15].

Human MAPL has a BAM (beside a membrane)/GIDE (growth inhibition death E3 ligase) domain located between two transmembrane domains topologically oriented such that both N- and C-termini reside in the cytosol while the BAM domain resides in the mitochondrial intermembrane space (IMS) [10,11,14]. BAM domain proteins are found in every domain of life (eukaryotes, bacteria, and archaea) and its patchy distribution has been explained by several independent horizontal gene transfer (HGT) events [7]. In human MAPL, a C-terminal cytosolic RING finger domain is present following the last transmembrane domain. Most studies have focused on the function of this RING domain. In the case of its role in regulating mitochondrial dynamics, MAPL stabilizes Drp1 via SUMOylation [9] and promotes the degradation of Mfn via ubiquitylation [5]. Hence, upregulation of MAPL results in small fragmented mitochondria [5,6,9]; whereas depletion of MAPL results in elongated mitochondria [6]. The substrates of MAPL’s RING domain when involved in other cellular processes remain unknown; however, at least four different E2 conjugating enzymes as MAPL interacting partners suggesting the existence of several other substrates [13].

Compared to the RING domain, very little is known about the function of MAPL’s BAM domain. Previous work has shown that only the BAM domain is required for proper packaging of MAPL into MDVs trafficked to peroxisomes [8–10]. Due to their endosymbiotic origins it is commonly held that the mitochondrial membranes are distinct from those of the membrane trafficking system and do not participate in vesicular trafficking. However, recent evidence from experiments in human tissue culture cells suggests there are at least two pathways in which mitochondrial membranes are trafficked to other organelles via MDVs [10,7,16,17]. One pathway directs MDVs to lysosomes/multivesicular bodies in a PINK1-dependent manner [18], whereas another pathway that directs MAPL-containing MDVs to peroxisomes is retromer-dependent [8]. It is important to note that MDVs have not been reported in any organism other than humans and the extent to which MDVs can be generalized to other model systems is unknown. Unfortunately the proteins/protein complexes identified as required for MDV formation (PINK1 and retromer) have several other cellular functions and cannot be used as markers for the presence of MDVs. MAPL has been described as a specific cargo in MDVs trafficked to the peroxisome and represents the best candidate for tracking MDVs in other organisms [10]. The only other published study [12] focusing on MAPL’s BAM domain suggests that it is involved in a mitochondrial stress response pathway. This study shows that, in the presence of hydrogen peroxide, IMS-located Omi/HtrA2 protease cleaves the BAM domain of MAPL resulting in the degradation of the protein [12]. Inactivation of Omi/HtrA2 results in increased levels of mitophagy due to the increased levels of MAPL. A connection between Omi/HtrA2-dependent cleavage of MAPL and its delivery to peroxisomes has yet to be tested.

The potential homology of MDVs and bacterial OMVs (outer membrane vesicles) has not gone unnoticed [7,16,19]. Bacterial OMVs are important for nutrient acquisition, biofilm development, and pathogenesis [20]. While it is attractive to assume that these two processes are homologous (especially in light of the presence of MAPL homologues in prokaryotes), this avenue has not yet been investigated, as the machinery required for OMV production in bacteria is still unknown [20]. Only by examining MAPL function in other organisms can we begin to gain insight into the ancient conserved function of MAPL. In this study we searched for MAPL homologues in diverse eukaryotes in order to gain insight into the origins and ancient conserved function of MAPL.

## Materials and Methods

### Genome databases

Publicly available genomes were obtained from various online resources including the Joint Genome Institute, the Broad institute (http://www.broadinstitute.org/), the National Center for Biotechnology Information (NCBI), and some private websites. A list of genomes used in this study is included in S1 Table.

### Homology searching

BAM domain containing proteins were retrieved using a combination of BLAST [21] and HMMer (http://hmmer.janelia.org/) searching strategies of both the NCBI non-redundant database as well as individual genomes. Each putative MAPL homologue was assessed for domain structure using the Pfam database (http://pfam.xfam.org/). Only sequences that contained recognizable BAM/GIDE domains were retained for phylogenetic analysis. Some BAM/GIDE proteins were not present in predicted proteomes were reconstructed from genomic assembly sequences or EST sequence reads available from NCBI. Sequences retrieved in this study are listed in S2 Table.

### Phylogenetic analysis

BAM domain-containing proteins were aligned using MUSCLE [22] and manually adjusted as needed using Mesquite (http://mesquiteproject.org). Model testing was performed using ProtTestv1.3 [23] with a Gamma rate distribution and accounting for invariant sites as appropriate. Phylogenetic tree reconstructions were carried out using MrBayes v3.2.2 [24] for Bayesian analysis. Maximum likelihood bootstrap values were obtained using PhyML [25] and RaxML [26] with 100 pseudoreplicates using the LG [27] model. Species-specific duplications and long branches representing highly divergent sequences were removed from subsequent analyses in order to limit the effects of long-branch attraction.

## Results and Discussion

### MAPL-related proteins are present in basally diverging lineages of Viridiplantae, Holozoa and Fungi

**Fig 1:**
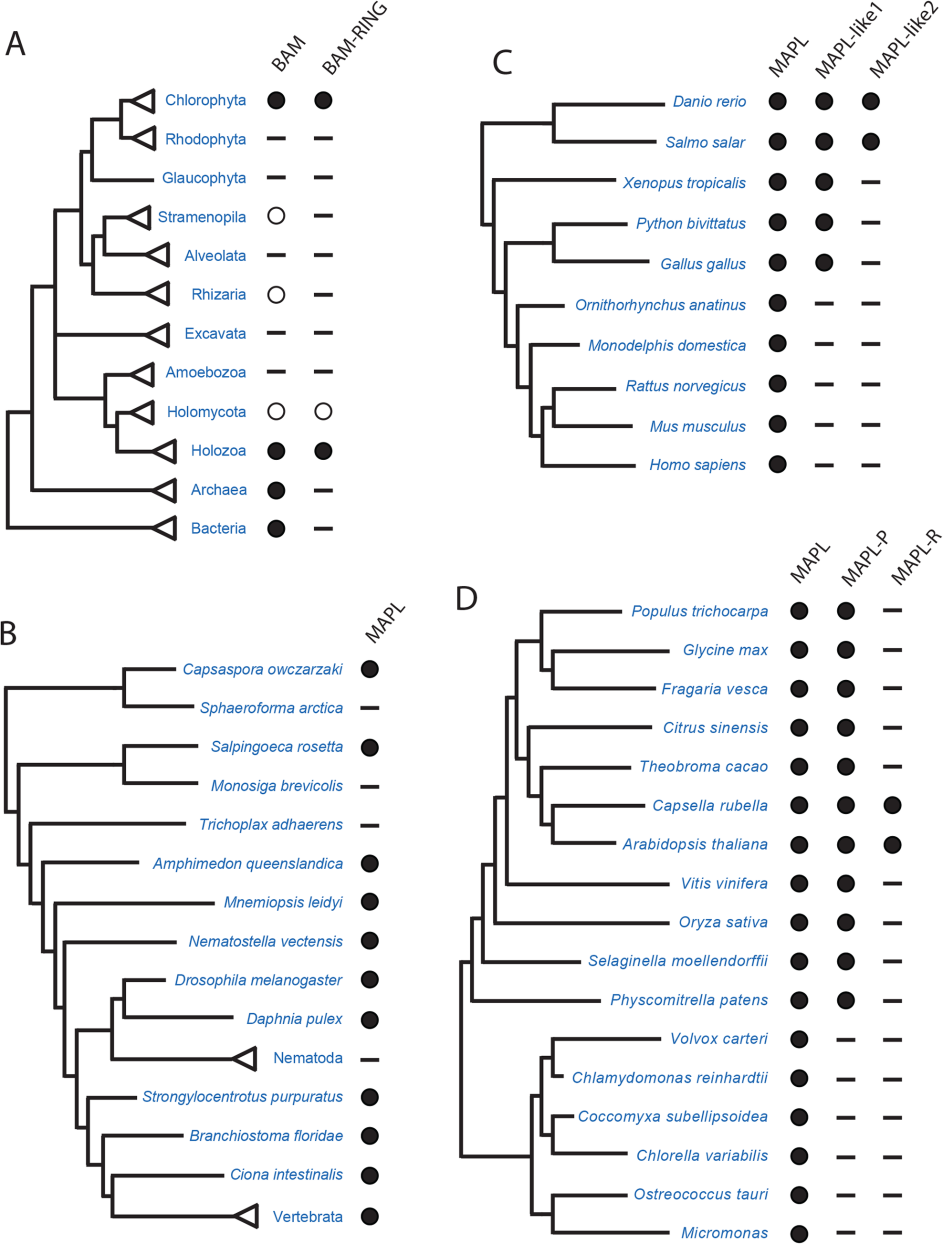
Distribution of BAM domain proteins across the tree of life. A. BAM domain distribution across the three domains of life. BAM domain proteins are present in all three domains of life, but only plants, animals, and a single fungus contain BAM proteins that are followed by a RING domain. Filled circles indicate many taxa contain at least one BAM protein. Open circles indicate only one or two species were identified with BAM proteins. B. Distribution of MAPL (BAM-RING) in holozoa (clade comprising animals and their closest single-celled relatives), with particular focus on non-vertebrates. Most species contain MAPL, but several instances of loss are recorded. C. Expansion of MAPL in the vertebrate lineage followed by loss in mammals. Multiple MAPL paralogues are present in non-mammalian vertebrates (MAPL2 and MAPL2-like). D. Expansion of MAPL in multicellular plants. Green algae contain a single MAPL whereas multicellular plants have gained a paralogue. The *Capsella*-*Arabidopsis* clade has further gained a paralogue that has lost the RING domain (MAPL-R).

We first searched for BAM domain proteins in representatives from diverse eukaryotic groups (Fig 1A). We identified putative MAPL homologues in the predicted proteomes of most metazoa (multicellular animals) as well as in the choanoflagellate *Salpingoeca rosetta* and the ichthyosporean *Capsaspora owczarzaki*, close single-celled relatives of animals. The presence of MAPL in organisms outside metazoa suggests an ancient origin of MAPL. In order to determine if MAPL was present in the ancestor of Opisthokonta we searched for homologues in fungi and identified MAPL-related proteins in only the chytridiomycete *Spizellomyces punctatus* and the neocallmastigomycete *Oprinomyces* sp. (Fig 1A). The *S*. *punctatus* BAM domain-containing protein lacked the characteristic C-terminal RING domain present in MAPL, but the protein in *Orpinomyces sp*. clearly contains a C-terminal RING domain. We thus conclude that metazoan MAPL with its extant domain structure likely descended from a protein that was present in the common ancestor of animals and fungi. However, the possibility of HGT of MAPL into these two fungal species cannot be ruled out by these data alone.

Although MAPL is present in numerous opisthokonts it is noticeably absent from several lineages. We could find no evidence for MAPL in any sequenced nematode species, or any fungal lineage outside those already mentioned. Although MAPL was present in one species of filasteria (*C*. *owczarzaki*) and one species of choanoflagellate (*S*. *rosetta*) MAPL could not be identified in sequences of genomes from the other sequenced holozoan protists *Sphaeroforma arctica* or *Monosiga brevicolis*. These absences suggest that although MAPL has been retained in diverse lineages, it has been repeatedly lost over the evolutionary history of the Opisthokonta.

Previous studies have shown that the fish *Salmo salar* contains two different MAPL paralogues [28,29]. Our investigation led to the discovery of a number of vertebrate species that contain MAPL paralogues (Fig 1C). This prompted a phylogenetic analysis (see below).

Although Andrade-Navarro et al. (2009) [7] found BAM domain-containing proteins in multicellular plants, the extent to which this protein is found in diverse eukaryotes was not investigated. We therefore searched for MAPL homologues in the predicted proteomes of members of the Archaeplastida (glaucophytes, red and green algae, and plants) and other protistan clades. We identified candidate proteins in all multicellular plants examined as well as several unicellular/colonial green algae including *Volvox carteri*, *Micromonas sp*., *Chlorella variabilis*, *Coccomyxa subellipsoidea*, *Ostreococcus tauri* and *Chlamydomonas reinhardtii* (Fig 1D). We also identified a plant-specific MAPL protein that we designated MAPL-P (Fig 1D and see below). Andrade-Navarro et al. [7] reported that proteins that lacked the C-terminal RING domain could be found in many plants; however, we found that the majority of archaeplastid BAM domain-containing proteins retained the C-terminal RING domain. Of the genomes investigated, only *Capsella rubella* and *Arabidopsis thaliana* contained proteins lacking a C-terminal RING domain. Although 33 diverse protist genomes were searched, BAM domains were identified in only two organisms outside the Opisthokonta and Viridiplanta, the rhizarian *Bigelowiella natans* and the brown alga *Ectocarpus siliculosis* (See S2 Table for a list of organisms lacking detectable BAM domains). These two proteins lacked the characteristic C-terminal RING domain present in metazoan MAPL proteins.

### Opisthokont and archaeplastid MAPL likely derive from a common ancestral protein

**Fig 2:**
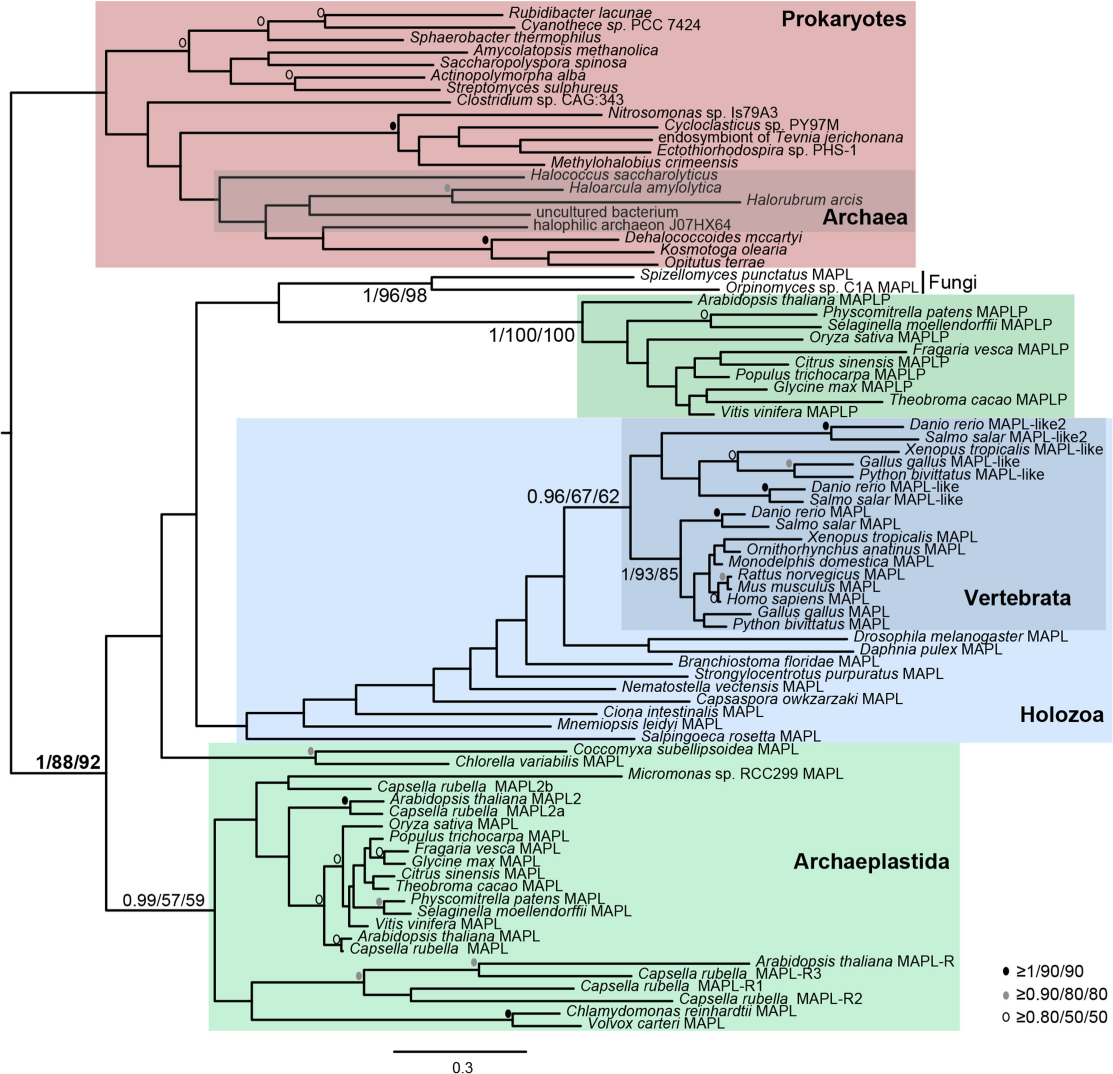
Phylogenetic reconstruction of BAM domain-containing proteins from opisthokonts, archaeplastida, and prokaryotes. BAM domain-containing protein sequences were aligned using MUSCLE, Sites that could not be aligned with confidence (including the eukaryote-specific RING domains) were removed manually. The resulting alignment was subjected to phylogenetic analysis (see methods section for details). In this analysis, prokaryotic BAM proteins group together to the exclusion of all eukaryote proteins. Thus, the BAM domain-containing proteins present in various eukaryotes cannot be traced to independent HGT events. In this and all following phylogenetic analyses, numerical values represent Bayesian posterior probabilities and maximum-likelihood bootstrap values (Bayesian/PhyML/RAxML). Node values are given to highlight the clades of interest, denoted by coloured boxes and annotated by protein name. All other node support is iconized as inset.

Andrade-Navarro et al. [7] suggested that BAM domains in various organisms are derived from several independent HGT events. If this were the case, a phylogenetic reconstruction would reveal distinct relationships between certain prokaryotic and eukaryotic proteins. In order to assess the relationships of eukaryotic BAM proteins we performed a phylogenetic analysis on all putative MAPL proteins along with BAM protein sequences from diverse bacterial and archaeal species (Fig 2). It is important to note that no prokaryotic sequence contained a C-terminal RING domain (Fig 1) and thus the RING domain was not included in the phylogenetic analysis.

Our analysis revealed that prokaryotic BAM proteins form a robustly supported clade to the exclusion of all opisthokont and archaeplastid MAPL proteins. The plant-specific clade designated as MAPL-P formed a robust clade with no obvious affinity to the other plant sequences. The *A*. *thaliana* and *C*. *rubella* proteins that lack the C-terminal RING domain (MAPL-R) group weakly with other archaeplastid MAPL proteins but strongly group within the larger eukaryotic clade (Fig 2). This suggests that HGT events are not responsible for the presence of the MAPL-R proteins in the Archaeplastida. The lack of the RING domain can instead be attributed to secondary loss.

### Independent expansion of MAPL in multicellular plants and animals

When searching for MAPL homologues in diverse eukaryotes, we noticed that several genomes contained more than one BAM-RING protein. Several vertebrate genomes contained two distinct MAPL proteins while several plant genomes contained many BAM domain-containing proteins. In order to identify lineage-specific duplications we reconstructed the phylogenies of these proteins in Viridiplantae and Opisthokonta.

**Fig 3:**
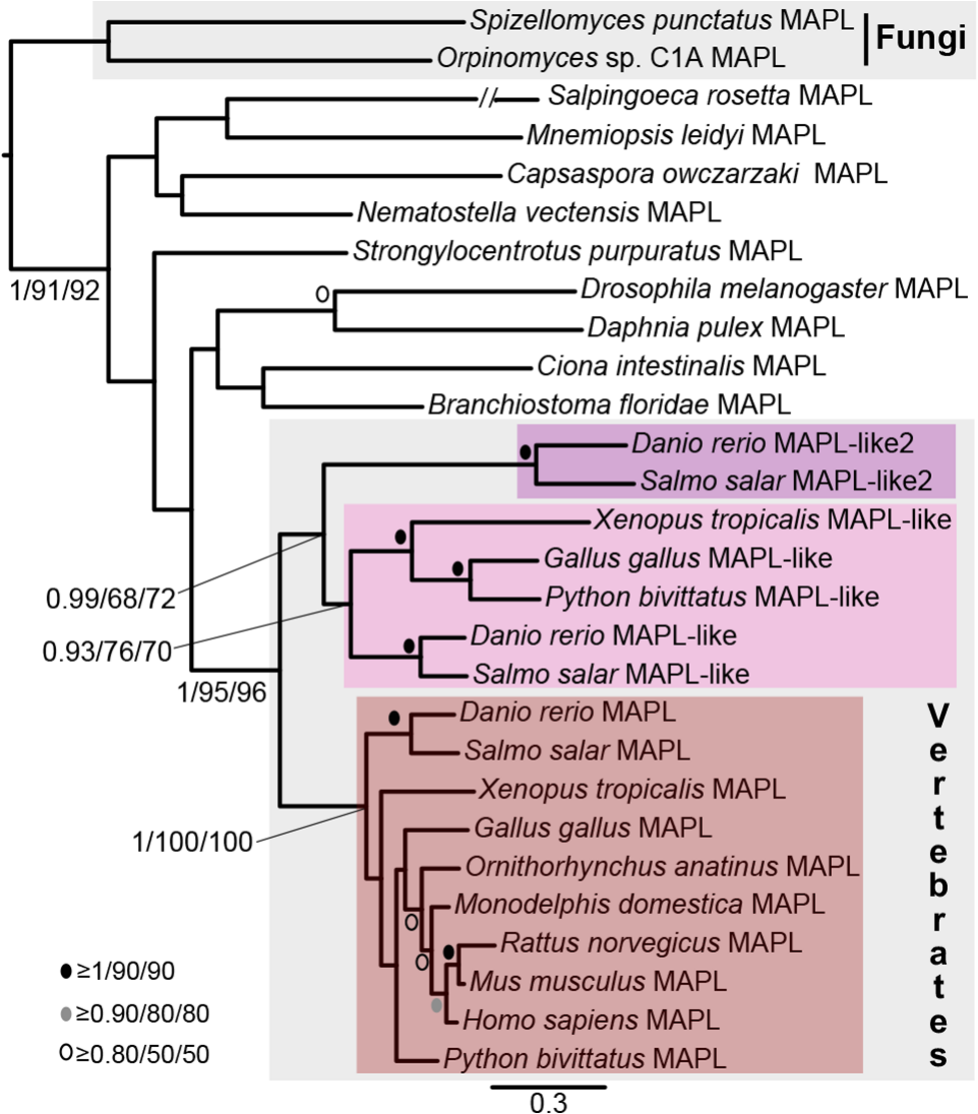
Phylogenetic analysis of Opisthokont MAPL proteins. This analysis demonstrates that all vertebrate MAPL proteins group together to the exclusion of all other opisthokont MAPL proteins. MAPL has been retained in all major vertebrate clades. MAPL-like is an ancient vertebrate protein lost in the mammalian lineage. MAPL-like2 is specific to fishes. The RING domain was included in the alignment in this analysis as the vast majority of predicted proteins contained this domain. Node support as in Fig 2.

Within the vertebrates we found that, while mammals contain only a single MAPL protein, amphibians and reptiles contain two MAPL paralogues while the fishes *Danio rerio* and *S*. *salar* contained three and two paralogues, respectively (See Tacchi et al. [28,29]). The apparent discordance between the number of paralogues in the two fishes compelled us to search for the potential missing *S*. *salar* sequence. In our searches of the *S*. *salar* EST database we found a third MAPL transcript that encodes a protein similar to the third *D*. *rerio* sequence. Our reconstructed phylogeny demonstrates that there was likely a duplication of MAPL at the base of vertebrates producing two proteins, MAPL and MAPL-like, one of which (MAPL-like) was subsequently lost in the mammalian lineage (Fig 3). These data also demonstrate that another independent duplication occurred in the fish lineage producing MAPL-like2 in fishes. It is interesting to note that MAPL and MAPL-like2 (the fish-specific paralogue) have been studied in *S*. *salar*, but the function of MAPL-like has not yet been investigated [28, 29].

**Fig 4:**
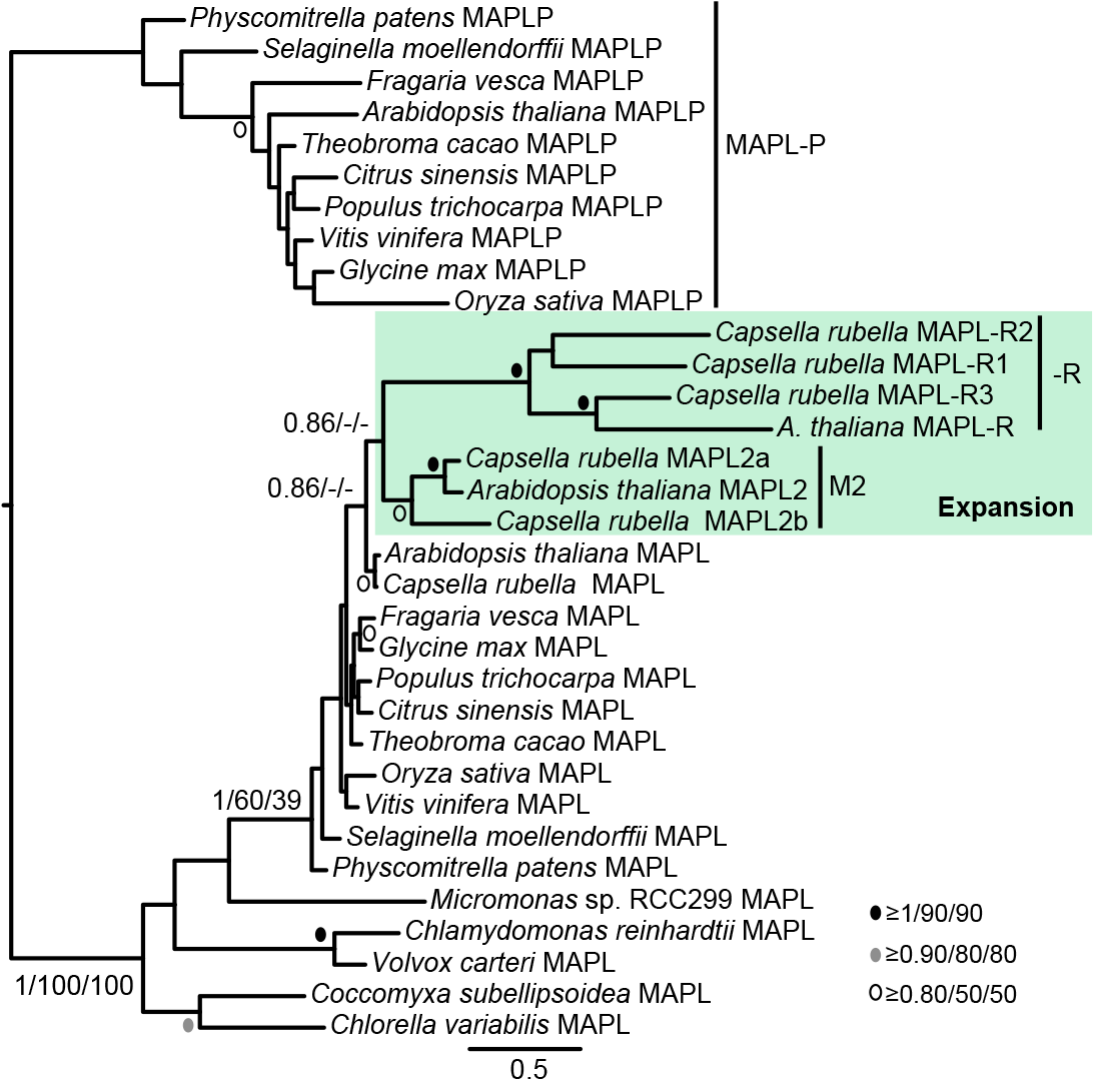
Phylogenetic analysis of Archaeplastid MAPL proteins. This analysis demonstrates that *A*. *thaliana* and *C*. *rubella* BAM proteins that lack the RING domain group within a weakly supported clade comprising sequences from multicellular plants that retain a RING domain. The RING domain was excluded from this analysis. Node support as in Fig 2.

Within Archaeplastida, multicellular plants contain very closely related paralogues of MAPL proteins. Most plant MAPL proteins fall into one of two clades, one rather divergent clade specific to multicellular plants (long branch MAPL-P clade in Fig 2) and a less divergent clade that shares features with the MAPL proteins found in green algae. While most of the proteins that we identified contained C-terminal RING domains, some of the paralogues identified in *Arabidopsis thaliana* and the closely related species *Capsella rubella* lacked the C-terminal domain. Our phylogenetic analysis of Archaplastid MAPL (Fig 4) further indicates that MAPL-R proteins represent a divergent lineage-specific expansion of MAPL in the *Arabidopsis*/*Capsella* clade that has subsequently lost the C-terminal RING domain.

### Stramenopile and rhizarian BAM/GIDE proteins are related to bacterial proteins

**Fig 5:**
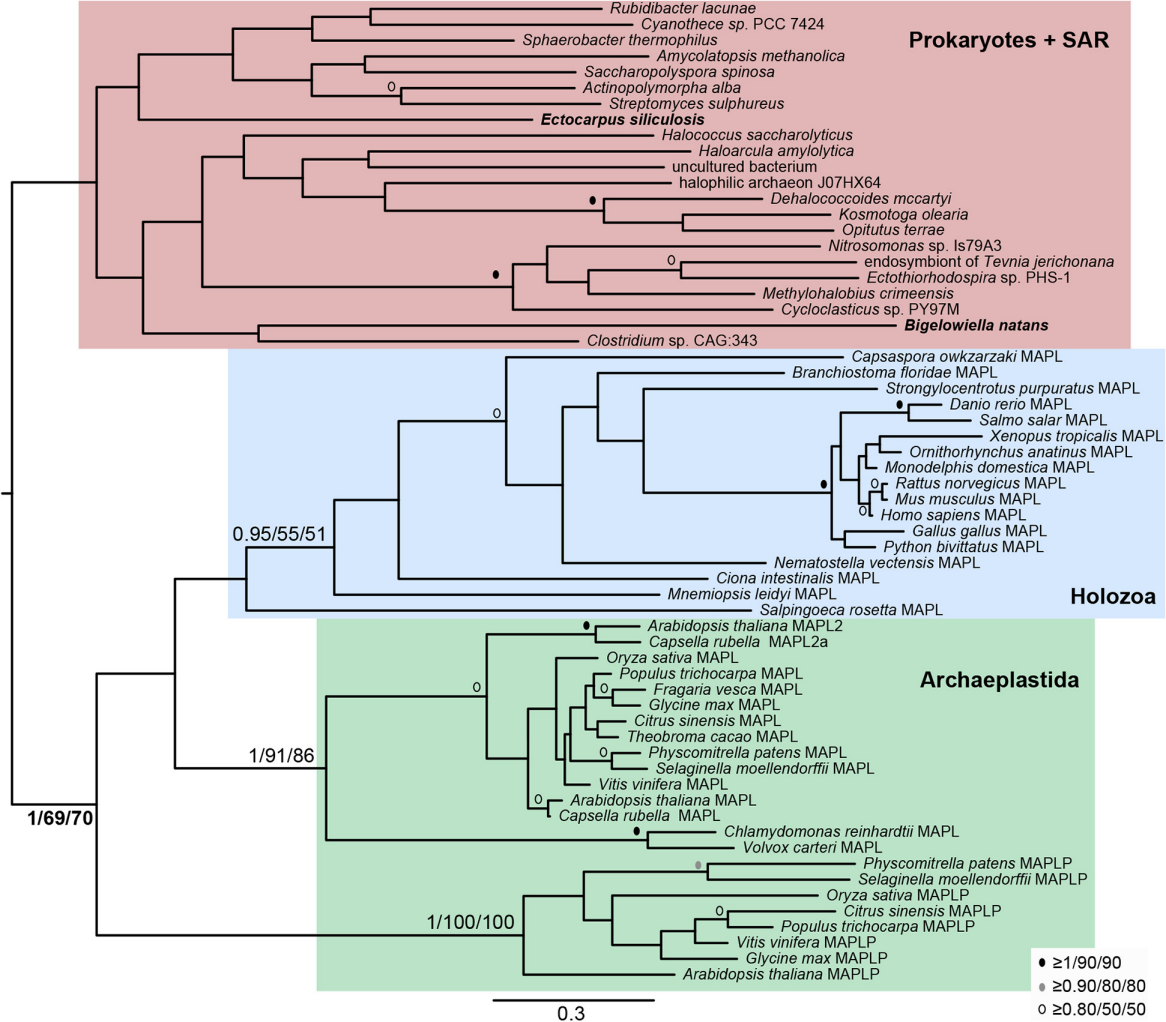
Phylogenetic analysis of SAR BAM proteins. In this analysis the BAM proteins from *E*. *siliculosis* and *B*. *natans* group with prokaryotic sequences suggesting that these eukaryotic proteins have a different origin than other MAPL and might be derived from recent HGT events. The RING domain was excluded from this analysis. Analysis and node support as in Fig 2.

In order to determine if the BAM proteins identified in representatives from the SAR (stramenopiles, alveolates, rhizaria) clade are more closely related to other eukaryote sequences or prokaryote sequences, BAM protein sequences from *B*. *natans* and *E*. *siliculosis* were added to our previous alignment used to generate Fig 2 but with all long-branches and lineage-specific duplications removed. To our surprise, we found that the SAR BAM domain proteins were excluded from the other eukaryote sequences (Fig 5). This suggests that recent HGT events might be responsible for the presence of BAM domains in these two species alone.

### Functional and evolutionary implications

Presence of conserved BAM-RING domain architecture in diverse eukaryotes and the relative rarity of loss of the RING domain suggests that both domains are important for the overall function of MAPL. If the domains functioned independently it would be expected that the domains would be found separated from one another more frequently over the course of eukaryote evolution. This is interesting because studies to date have not found a link between the two domains and the different functions discovered in animals seem to be distinct.

Duplication and divergence in some lineages, like vertebrates and plants, suggests that functional specialization has occurred. The duplication at the base of vertebrates is of particular interest because the divergent paralogue is lost in mammals marking a relatively rare example of secondary loss in the mammalian lineage. Investigation of MAPL-like proteins in model fish, reptile, and amphibian species will be of great interest for understanding the conserved function of MAPL in mammalian cells.

Although eukaryotic MAPL appears to derive from a single eukaryotic protein with similar domain architecture to extant MAPL, the ancestry of MAPL remains difficult to resolve. Previous work [7] suggested that MAPL may have arisen in bilateria and was subsequently horizontally transferred to plants and prokaryotes. Our discovery of MAPL in fungi, unicellular holozoa and green algae makes this suggestion unlikely. Since only archaeplastids and opisthokonts retain MAPL it is impossible to determine if the protein was present in the LECA (last eukaryotic common ancestor) or if MAPL was horizontally transferred very early between the opisthokont and viridiplantae lineages. Although unlikely, it is conceivable that MAPL might have evolved in either the ancestor of all opisthokonts or the ancestor of all viridiplantae and a single HGT event between them could account for the current distribution of the protein. We feel that a more parsimonious explanation is that MAPL is ancient and was present in the LECA (perhaps by HGT from bacteria or even the mitochondrial endosymbiont) but was independently lost in several eukaryotic lineages while retained in Opisthokonta and Viridiplantae.

## Conclusions

MAPL is involved in organelle dynamics, mitophagy, mitochondria to nucleus signaling, and is packaged as cargo in MDVs destined for peroxisomes. The presence of MAPL in diverse eukaryotes allows us to infer its ancient origins, but several questions still remain. Specifically, although MAPL is ancient we currently have no way of knowing if it was present in the LECA or if it arose later in evolution. It is also still unclear how many HGT events may have occurred between prokaryotes.

Its taxonomic distribution suggests that MAPL functions in several single-celled eukaryotes. As multicellular animals are cluttered with proteins and pathways required for obligate multicellular life, investigation into MAPL function in single-celled organisms like the choanoflagellate *S*. *rosetta*, the filasterian *C*. *owczarzaki*, and the green alga *C*. *reinhardtii* is critical to understanding MAPL’s ancestral function. We hope that our study provides the impetus for future functional work on MAPL in organisms outside metazoa.

## Supporting Information

S1 Table Genome databases used in this study and indication of BAM presence or absence. (XLSX)

S2 Table Proteins retrieved in this study. (XLSX)
